# A rare cause of a common symptom, Anakinra is effective in the urticaria of Schnitzler Syndrome: a case report

**DOI:** 10.1186/1757-1626-1-348

**Published:** 2008-11-24

**Authors:** Lisa A Devlin, Gary Wright, J David M Edgar

**Affiliations:** 1 Regional Immunology Service, Royal Hospitals, The Belfast Trust, Grosvenor Road, Belfast BT12 6BN; 2 Department of Rheumatology, Royal Hospitals, The Belfast Trust, Grosvenor Road, Belfast BT12 6BN

## Abstract

**Introduction:**

Schnitzler Syndrome is an uncommon, inflammatory condition that presents with a constellation of chronic unremitting urticaria, fever, bone pain, arthralgia or arthritis, and a monoclonal IgM gammopathy. There is usually neutrophilia and raised inflammatory markers. Delayed diagnosis is common and treatment often unsuccessful.

**Case presentation:**

We report the case of a 43-year-old caucasian man who presented with urticaria unresponsive to conventional therapy. There was considerable delay in recognition of this as Schnitzler Syndrome, and symptoms were unresponsive to conventional immunosuppressive therapy.

Commencement of anakinra was associated with a rapid and sustained clinical response.

**Conclusion:**

Schnitzler Syndrome is a rare disorder that mimics chronic idiopathic urticaria. This diagnosis should be considered in patients with urticaria unresponsive to antihistamines and conventional immunosuppressive therapy. Anakinra is an effective treatment although further studies are required, to determine long term therapeutic requirements and assess any potential adverse effects.

## Introduction

Chronic idiopathic urticaria (CIU) is a common, benign condition for which patients are frequently referred to allergy or dermatology clinics. The investigation and management of patients with urticaria has recently been reviewed [1]. The majority of patients presenting with urticaria do not require extensive laboratory investigation and their symptoms usually respond to regular oral antihistamines, albeit often at high dose. For selected unresponsive patients, the use of immunosuppressive agents is occasionally required. The case we report initially presented with an urticarial rash only, but this was later followed by the development of fever, arthralgia, lymphadenopathy and raised inflammatory markers. Extensive investigation was therefore undertaken and a provisional diagnosis of Adult Onset Still's Disease (AOSD) made. However in view of the lack of response to immunosuppressive treatment and the subsequent dramatic response to anakinra, the final diagnosis of Schnitzler Syndrome (SS) was established. SS is a rare condition, with less than 100 cases described. Because of its rarity and the lack of a specific diagnostic test, it is likely to be under diagnosed. This case highlights its importance as a differential diagnosis of CIU and its apparent exquisite sensitivity to treatment with anakinra.

## Case presentation

**Figure 1:**
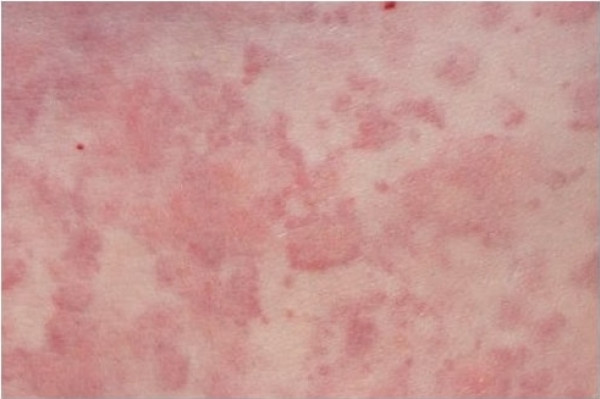
Urticarial rash at presentation.

A previously well 43-year-old man presented with chronic urticaria (Figure 1). There was no obvious precipitant identified from the history, he was systemically well and investigations including FBP, U&E, LFTs, TFTs, ESR, and CRP were normal. A polyclonal increase in IgG and IgA was noted with an associated small (< 1 g/L) IgM kappa paraprotein. The rash was resistant to conventional treatment with non-sedating antihistamines.

At review, 6 months later, he had developed generalized fatigue, fever, night sweats, arthralgia, right knee and tibial pain, weight loss and bilateral axillary and inguinal lymphadenopathy.

Investigations showed a persistent neutrophilia (> 15 × 10^9^/L) and raised ESR and CRP. Other haematological indices were within normal limits. A persistent polyclonal elevation in IgG, A and M was noted, with the IgM kappa paraprotein increased to 3.3 g/L. Serum ferritin was mildly elevated (361 μg/L, normal range 18–325). An inflammatory arthritis was suspected, and admission arranged for further investigation and treatment.

**Figure 2:**
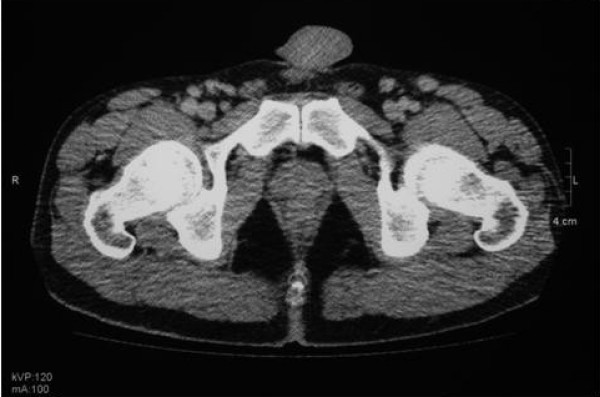
CT scan of pelvis indicating multiple enlarged lymph nodes.

Quotidian spiking fevers (> 38°C) were recorded whilst an inpatient. Isotope bone scan showed increased tracer uptake at proximal aspect of right tibia in comparison with the left. MRI right knee showed non specific marrow signal change at distal end of femur/proximal tibia. CT chest, abdomen and pelvis showed florid bilateral axillary and inguinal lymphadenopathy (Figure 2). Serum antibodies to nuclei, extractable nuclear antigens, double stranded DNA, cyclic citrullinated peptide, and neutrophil cytoplasmic antigens were all negative. Rheumatoid factor was negative and serum C3 and C4 levels were normal. There was a slight elevation in B2 microglobulin and plasma viscosity, but lymphocyte subsets including kappa/lambda ratios were normal, and urine analysis was negative for Bence Jones protein. Serological investigations for EBV, CMV, HIV, syphilis, hepatitis A, B and C, borrelia burgdoderi, brucella abortus, chlamydia, Q fever, mycoplasma, and HHV8 were negative. ASOT was < 200. Urine cultures for mycobacterium were repeatedly negative.

**Figure 3:**
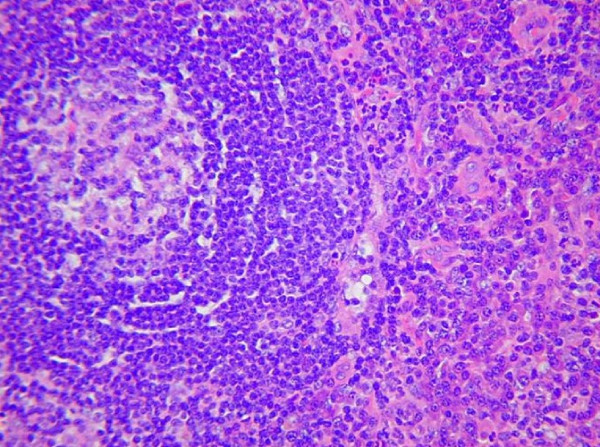
Lymph node biopsy demonstrates widespread infiltration by plasma cells.

Skin biopsy demonstrated an inflammatory infiltrate in the epidermis, suggestive of either rheumatoid arthritis or Still's disease. Lymph node biopsy revealed polyclonal reactive hyperplasia with abundance of plasma cells (Figure 3). Castleman disease was considered but excluded after a further biopsy and review by several expert histopathologists. Bone marrow biopsy showed reactive changes only.

Mutations were not found in the coding regions of MVK, TNFRSF1A, and NLRP3 (previously NALP3/CIAS1) making the diagnoses of Hyper IgD syndrome, TNF receptor associated periodic syndrome (TRAPS), and Muckle Wells/Familial Cold Urticaria all unlikely.

A tentative diagnosis of Adult Onset Still's Disease (AOSD) was made. Trials of etoricoxib, and subsequent oral prednisolone (40 mg daily) with methotrexate (20 mg weekly) were however ineffective. Two cycles of pulsed methylprednisolone (250 mg and 500 mg) resulted in transient improvement in both symptoms and inflammatory markers over the initial 36 hours, but symptoms returned on completion of cycle. A trial of infliximab (5 mg/kg) resulted in a paradoxical exacerbation with night sweats, fever, arthralgia and urticaria.

Symptoms and signs persisted over the next year despite symptomatic treatment. The patient was severely disabled by these and only able to work part time.

Four years after initial presentation, a further trial of prednisolone (60 mg/day) was commenced. 48 hours after commencement of treatment there was almost complete resolution of symptoms. Initial CRP (149 mg/L) fell to 10.8 mg/L after 13 days treatment. Azathioprine was commenced as a steroid sparing agent however the development of severe arthralgia, joint stiffness, rash, and fever with a CRP of 164 mg/L led to its withdrawal after 9 days. Mycophenolate mofetil (1 g BD) was substituted however despite 3 months treatment; prednisolone dose could not be reduced below 50 mg/day.

Given the unresponsiveness of this patient's disease to a number of immunosuppressant drugs, and reports of a beneficial response in refractory AOSD [2], a trial of anakinra, (100 mg/day) was commenced. Within 24 hours there was almost complete resolution of symptoms. CRP normalized within 7 days. The patient has remained on anakinra therapy for 16 months with continued symptom control. Temporary interruption of treatment on two occasions was associated with recurrence of rash, fever and arthralgia within 24 hours. Symptoms quickly resolved on recommencing anakinra. In light of the clinical course and following an extensive literature review, the final diagnosis of SS was made. The patient fulfilled both essential criteria and 6 of the additional criteria established by Lipsker et al (Table 1) [3].

**Table 1:** Diagnostic Criteria for Schnitzler Syndrome*

Urticarial skin rash, monoclonal IgM component, and at least 2 of the following criteria:
Fever
Arthralgia or arthritis
Bone pain
Lymphadenopathy
Hepato- and/or splenomegaly
Elevated ESR
Leucocytosis
Abnormal findings on bone morphological investigations
*Another cause must be eliminated in all cases, most notably hyper IgD syndrome, AOSD, hypocomplementaemic urticarial vasculitis, acquired C1 inhibitor deficiency, and cryoglobulinaemia.

## Discussion

SS is an uncommon syndrome with only 94 cases reported in the literature [4]. It was first described by L. Schnitzler [5], as a clinical entity consisting of chronic urticaria, fever, bone pain, arthralgia or arthritis, and a monoclonal IgM gammopathy.

This patient was at first thought to have CIU but failed to respond to conventional therapy. The subsequent development of systemic symptoms led to a wide differential being considered and several specialists reviewed his case. Because of the prominence of joint symptoms, AOSD was the provisional diagnosis for some time. It is important to note that the patient met the diagnostic criteria for AOSD established by Yamaguchi et al and that these are not therefore entirely specific and potential remains for diagnostic confusion between the two conditions [6]. Specific diagnostic tests do not exist for either AOSD or SS, however serum ferritin levels detected in AOSD are usually higher than in this patient and the presence of a paraprotein (typically IgM kappa) is characteristic of SS. Skin, lymph node and bone marrow biopsies in SS often show non specific 'reactive changes' on histopathology.

SS is a rare chronic condition and there are no reports of spontaneous remission [3]. The mean age of onset is 51 years, diagnostic delay is common and greater than 5 years in most cases [3]. In the recent review of 94 published cases, the prognosis was regarded as favorable with 91% survival after 15 years [4]. A significant long term complication (15%) remains the development of lymphoproliferative disease, especially Waldenstroms macroglobulinaemia 10–20 years following the onset of symptoms [3,4]. 3 patients developed amyloidosis in that series [4].

Chronic urticaria, unresponsive to antihistamines is characteristic of SS and is an important clinical message of this case. Oral steroids have not been beneficial at acceptable doses, and given the chronicity of the disease, are associated with significant cumulative side effects [4]. Despite the significant evidence of inflammation in this condition, there is almost complete failure to respond to a variety of immunosuppressants including azathioprine, ciclosporin, methotrexate, high dose immunoglobulin, hydroxychloroquine, dapsone, colchicine, rituximab, mycophenolate and infliximab [7-10]. Until recently, therefore, treatment of SS has been associated with a very disappointing outcome.

Recently, anakinra, an IL-1 receptor antagonist has been associated with an excellent outcome [7-10]. Within 24 hours of commencing anakinra, our patient had almost complete resolution of his symptoms, and has since been able to return to work full time. Discontinuation of anakinra for 1 day, on two occasions, was associated with a rapid disabling return of symptoms.

Six patients with SS have to date made a complete response, both symptomatically, and with a reduction in inflammatory markers and white cell count, to anakinra [7-10]. The longest published follow up is 18 months, our patient has been treated for 21 months and continues to require therapy. No patient has to our knowledge discontinued anakinra and remained in remission. It therefore appears likely that long term treatment will be required, however periodic withdrawal of therapy seems reasonable to assess current disease activity.

Anakinra has been well tolerated, with no significant side effects in either our patient or other cases of SS reported to date. One patient developed a mild reaction at the injection site which resolved after few weeks [8], and one patient had an injection associated headache [10], which subsided with ongoing therapy.

The pathogenesis of SS is essentially unknown. The reported universal response to anakinra suggests a key role of IL-1 in the pathophysiology.

Interestingly, anakinra has also been reported to provide beneficial effects in patients with Muckle Wells syndrome [11]. Given that there is also considerable overlap in the clinical features of SS and Muckle Wells syndrome, this raises the possibility of common pathophysiology. One published case of SS had molecular analysis carried out for mutations in the coding region of NALP3 (the defect associated with Muckle Wells syndrome), but no mutations were identified [7]. With increasing recognition and characterization of patients with SS, the possibility that it forms part of the spectrum of cryopyrin diseases remains.

## Conclusion

SS is a rare cause of a common symptom (urticaria), but this typically occurs in patients with other systemic symptoms. These systemic symptoms show considerable overlap with AOSD and therefore immunologists, dermatologists and rheumatologists all need to be aware of this potential diagnosis. The presence of an IgM paraprotein is an important indicator in favour of a diagnosis of SS.

Patients with SS have a significant risk of late malignancy and should be carefully monitored for its development. Until now, treatment has largely been unsuccessful, however the introduction of anakinra has been very effective in this and 4 other published cases. Our experience suggests that treatment with anakinra is likely to be required in the long term in order to control symptoms, reduce inflammation and diminish the risk of long term side effects.

## Competing interests

The authors declare that they have no competing interests.

## Authors' contributions

LAD collated clinical data and drafted the initial manuscript. JDME provided clinical data, revised and amended the manuscript. GW provided clinical data and reviewed the manuscript. All authors have read and approved the final manuscript.

## Consent

Written informed consent was obtained from the patient for publication of this case report and accompanying images. A copy of the written consent is available for review by the Editor-in-Chief of this journal
